# Supplementary material for: Isolation, Characterization, Genome Annotation, and Evaluation of Hyaluronidase Inhibitory Activity in Secondary Metabolites of Brevibacillus sp. JNUCC 41: A Comprehensive Analysis through Molecular Docking and Molecular Dynamics Simulation
Source: Int J Mol Sci. 2024 Apr 23;25(9):4611. doi: 10.3390/ijms25094611 (PMC11083829; doi:10.3390/ijms25094611)

Isolation, Characterization, Genome Annotation, and Evaluation of Hyaluronidase Inhibitory Activity in Secondary Metabolites of *Brevibacillus* sp. JNUCC 41: A Comprehensive Analysis through Molecular Docking and Molecular Dynamics Simulation

Yang Xu [1], Xuhui Liang[1] and Chang-Gu Hyun *

Department of Chemistry and Cosmetics, Jeju National University, Jeju 63243, Korea.
*Correspondence: cghyun@jejunu.ac.kr; Tel.: +82-64-754-1803

Supplementary materials

Table S1. COG functional gene classification of the genome 2

Table S2. A list of genetic determinants of CAZy detected in the chromosome of *Brevibacillus* sp. JNUCC 41 3

Table S3. A list of genetic determinants of virulence (VFs) detected in the chromosome of *Brevibacillus* sp. JNUCC 41 5

Table S4. ADMET properties of compounds 6

Table S5. Drug-likeness properties of compounds 7

Figure S1. ^{1}H NMR of compound 1 8

Figure S2. ^{13}C NMR of compound 1 9

Figure S3. ^{1}H NMR of compound 2 10

Figure S4. ^{13}C NMR of compound 2 11

Figure S5. ^{1}H NMR of compound 3 12

Figure S6. ^{13}C NMR of compound 3 13

Figure S7. ^{1}H NMR of compound 4 14

Figure S8. ^{13}C NMR of compound 4 15

Figure S9. ^{1}H NMR of compound 5 16

Figure S10. ^{13}C NMR of compound 5 17

Table S1. COG functional gene classification of the genome

COG_class	Description	Number
J	Translation, ribosomal structure and biogenesis	290
A	RNA processing and modification	0
K	Transcription	408
L	Replication, recombination and repair	152
B	Chromatin structure and dynamics	0
D	Cell cycle control, cell division, chromosome partitioning	236
Y	Nuclear structure	0
V	Defense mechanisms	116
T	Signal transduction mechanisms	291
M	Cell wall/membrane/envelope biogenesis	256
N	Cell motility	47
Z	Cytoskeleton	4
W	Extracellular structures	3
U	Intracellular trafficking, secretion, and vesicular transport	41
O	Posttranslational modification, protein turnover, chaperones	188
X	Mobilome: prophages, transposons	31
C	Energy production and conversion	250
G	Carbohydrate transport and metabolism	309
E	Amino acid transport and metabolism	513
F	Nucleotide transport and metabolism	142
H	Coenzyme transport and metabolism	273
I	Lipid transport and metabolism	274
P	Inorganic ion transport and metabolism	247
Q	Secondary metabolites biosynthesis, transport and catabolism	118
R	General function prediction only	397
S	Function unknown	220

Table S2. A list of genetic determinants of CAZy detected in the chromosome of *Brevibacillus* sp. JNUCC 41

Gene ID	**dbCAN_sub**	**Gene ID**	**dbCAN_sub**
QOS87884.1	GT2(7-176)	QOS90431.1	AA4(13-229)
QOS87908.1	AA4(3-228)	QOS90512.1	GT51(99-282)
QOS88030.1	GT51(62-236)	QOS90731.1	CE4(42-145)
QOS88137.1	CBM34(6-124)+GH13_20(173-469)	QOS91004.1	GH18(242-517)
QOS88138.1	GH13_45(65-358)	QOS91021.1	GT1(180-390)
QOS88139.1	GH13_31(28-372)	QOS91054.1	GT2(7-97)
QOS88211.1	CE1(15-237)	QOS91071.1	GT4(189-353)
QOS88245.1	GH1(2-466)	QOS91177.1	GH18(154-452)
QOS88275.1	CE3(31-225)	QOS91197.1	GT4(1033-1177)
QOS88374.1	CE4(82-204)	QOS91204.1	GH73(959-1089)
QOS88571.1	CE4(125-249)	QOS91214.1	AA1(98-488)
QOS88624.1	GH4(6-185)	QOS91215.1	GH8(65-417)
QOS88730.1	GH25(7-177)	QOS91235.1	GT28(202-349)
QOS88860.1	GH13_29(25-369)	QOS91252.1	CE4(269-389)
QOS88980.1	CE14(8-126)	QOS91284.1	GT4(257-413)
QOS89144.1	GH170(1-333)	QOS91377.1	GH18(131-408)
QOS89145.1	GH3(116-351)	QOS91399.1	GT51(60-236)
QOS89148.1	GH109(1-152)	QOS91471.1	GH13_31(28-376)
QOS89360.1	AA4(6-242)	QOS91582.1	GT4(184-326)

QOS89377.1	AA4(4-227)	QOS91583.1	GT26(59-227)
QOS89427.1	GH13_14(192-506)	QOS91586.1	GT4(203-354)
QOS89451.1	GT4(168-325)	QOS91590.1	GT2(38-162)
QOS89455.1	GT4(165-320)	QOS91684.1	GT51(62-238)
QOS89460.1	GT4(168-326)	QOS91706.1	CE14(8-126)
QOS89474.1	GT5(2-471)	QOS91766.1	GH13_5(31-371)
QOS89476.1	CBM48(33-116)+GH13_9(185-485)	QOS91945.1	CE4(51-175)
QOS89477.1	GH15(17-318)	QOS91995.1	CE9(11-383)
QOS89598.1	GT27(61-340)	QOS92067.1	GT51(84-264)
QOS89599.1	GT2(5-160)	QOS92210.1	GH179(19-334)
QOS89600.1	GT4(406-567)	QOS92211.1	GH179(19-342)
QOS89602.1	GT2(5-173)+GT2(248-408)	QOS92258.1	CBM50(29-70)
QOS89608.1	GH18(118-405)	QOS92305.1	CE4(56-180)
QOS89692.1	CE4(42-155)	QOS92331.1	AA7(21-250)
QOS89707.1	GT28(191-339)	QOS92425.1	AA6(2-169)
QOS89754.1	CE3(53-256)	QOS92473.1	GT4(256-362)
QOS89782.1	CE4(78-197)	QOS92609.1	CE4(34-143)
QOS89808.1	GH170(3-358)	QOS92619.1	GH3(115-338)
QOS89809.1	GH4(8-188)	QOS92655.1	CE4(117-243)
QOS89888.1	GT51(83-257)	QOS92740.1	GH23(105-206)
QOS89916.1	GT4(194-343)	QOS92746.1	GH179(18-206)

QOS89917.1	CE14(10-114)	QOS92800.1	CE4(37-160)
QOS90205.1	CE4(42-157)	QOS92805.1	AA6(5-195)
QOS90283.1	GT4(201-355)	QOS92828.1	GH171(51-408)
QOS90285.1	GT4(194-345)	QOS92853.1	GH14(24-415)+CBM20(435-520)

Table S3. A list of genetic determinants of virulence (VFs) detected in the chromosome of *Brevibacillus* sp. JNUCC 41

<table>
<tr><th>VFclass</th><th colspan="2">Virulence factors</th><th>JNUCC 41 (Prediction)</th></tr>
<tr><td rowspan="3">Adherence</td><td colspan="2">Fibronectin-binding protein (Listeria) (fbpA)</td><td>QOS88467.1</td></tr>
<tr><td colspan="2">LPS O-antigen (P. aeruginosa) (Pseudomonas)</td><td>QOS92532.1</td></tr>
<tr><td colspan="2">Streptococcal lipoprotein rotamase A(Streptococcus) (slrA)</td><td>QOS91396.1</td></tr>
<tr><td>Enzyme</td><td colspan="2">Serine-threonine phosphatase (Listeria) (stp)</td><td>QOS88478.1</td></tr>
<tr><td rowspan="8">Immune evasion</td><td colspan="2">Hyaluronic acid (HA) capsule (hasC)</td><td>QOS89457.1</td></tr>
<tr><td rowspan="5">Polyglutamic acid capsule</td><td>capA</td><td>QOS90628.1</td></tr>
<tr><td>capB</td><td>QOS90626.1</td></tr>
<tr><td>capC</td><td>QOS90627.1</td></tr>
<tr><td>capD</td><td>QOS89054.1; QOS90629.1; QOS92890.1</td></tr>
<tr><td>capE</td><td>QOS92939.1</td></tr>
<tr><td colspan="2">Polysaccharide capsule</td><td>QOS87883.1; QOS89453.1; QOS89456.1; QOS90270.1; QOS90281.1; QOS90284.1; QOS90288.1; QOS90290.1; QOS90291.1; QOS91196.1; QOS91283.1; QOS92531.1</td></tr>
<tr><td colspan="2">LOS(Campylobacter)</td><td>QOS91024.1</td></tr>
<tr><td rowspan="3">Regulation</td><td colspan="2">Carbon storage regulator A(Legionella) (csrA)</td><td>QOS91567.1</td></tr>
<tr><td colspan="2">CheA/CheY(Listeria) (cheY)</td><td>QOS88534.1</td></tr>
<tr><td colspan="2">LisR/LisK (Listeria) (lisR)</td><td>QOS91489.1</td></tr>
</table>

Secretion system	T6SS-II(Klebsiella) (clpB)	QOS88168.1
	TTSS (ysc-yop) secretion apparatus (Yersinia) (yscN)	QOS88524.1
Toxin	Hemolysin III (hlyIII)	QOS89682.1
	Cytolysin (Enterococcus) (cylR2)	QOS90796.1
Antiphagocytosis	Capsule (Enterococcus)	QOS88554.1
	Capsule (Klebsiella)	QOS92091.1
Iron uptake	ABC transporter (Corynebacterium) (fagC)	QOS87867.1
	Achromobactin biosynthesis and transport (Pseudomonas) (cbrD)	QOS88087.1
	Periplasmic binding protein-dependent ABC transport systems (Vibrio) (vctC)	QOS91480.1
	Pyoverdine (Pseudomonas) (pvdH)	QOS91765.1
Motility and export apparatus	Flagella (Campylobacter) (fliP)	QOS88536.1
Nutritional factor	Allantoin utilization (Klebsiella)	QOS87959.1
Other adhesion-related proteins	PDH-B (Mycoplasma) (pdhB)	QOS88033.1
Stress adaptation	Catalase (Neisseria) (katA)	QOS91358.1
Surface protein anchoring	Lipoprotein-specific signal peptidase II (Listeria) (lspA)	QOS88455.1

Table S4. ADMET properties of compounds

ADMET properties	Methyl indole-3-acetate	EGCG
**Absorption**		
Caco-2 permeability (cm/s)	-4.459	-6.717
P-gp inhibitor	No	Yes
P-gp substrate	No	Yes
Human intestinal absorption	94.739%	47.395%
**Distribution**		
Plasma protein binding	56.817%	88.242%
Volume distribution (L/kg)	0.976	0.485

Blood–brain barrier	Yes	No
**Metabolism**		
CYP1A2 inhibitor	Yes	No
CYP2C19 inhibitor	No	No
CYP2C9 inhibitor	No	No
CYP2D6 inhibitor	No	No
CYP3A4 inhibitor	No	Yes
**Elimination**		
Clearance rate (mL/min/kg)	10.460	14.450
$T_{1/2}$ (h)	0.898	0.496
**Toxicity**		
Hepatotoxicity	No	No
Ames toxicity	No	No
Skin sensitization	No	No
hERG inhibition	No	Yes
LD_{50} of acute toxicity (mol/kg)	2.314	2.522

LD_{50}: lethal dose; CYP: cytochrome p450; $T_{1/2}$: time required for plasma concentration of a drug to decrease by 50%; hERG: human Ether-a-go-go-Related Gene.

Table S5. Drug-likeness properties of compounds

**Drug-likeness properties**	**Methyl indole-3-acetate**	**EGCG**
MW	189.08	458.38
HBA	3	11
HBD	1	8
MR	54.16	112.06
RB	3	4
TPSA (Å^2)	42.090	225.060
Log *p*	2.200	1.893
Lipinski's Rule	Yes	No
Ghose Filter	Yes	Yes
Veber Filter	Yes	No
Egan Filter	Yes	No

MW: molecular weight; HBA: num. H-Bond acceptors; HBD: num. H-Bond donors; RB: num. rotatable bonds; MR: molar refractivity.

Figure S1. ^{1}H NMR of compound 1

Methyl indole-3-acetate

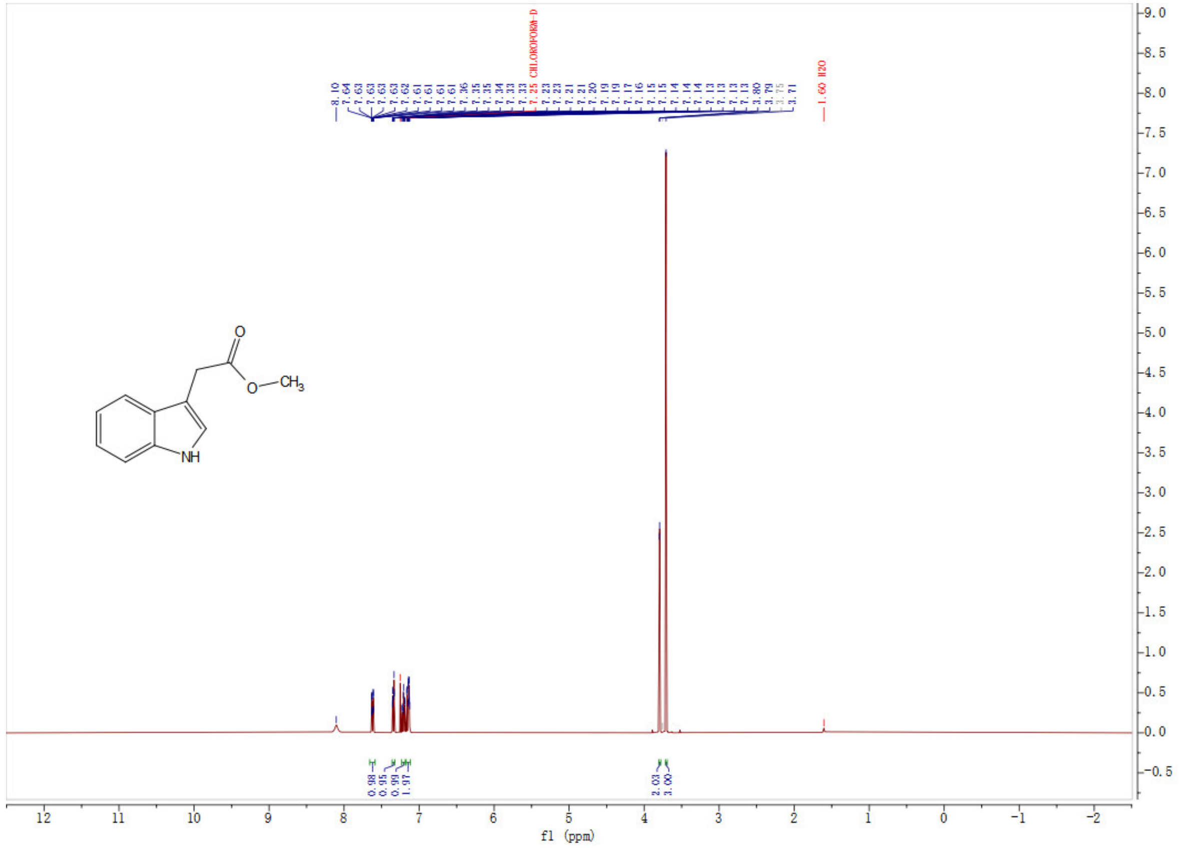

Figure S2. ^{13}C NMR of compound 1

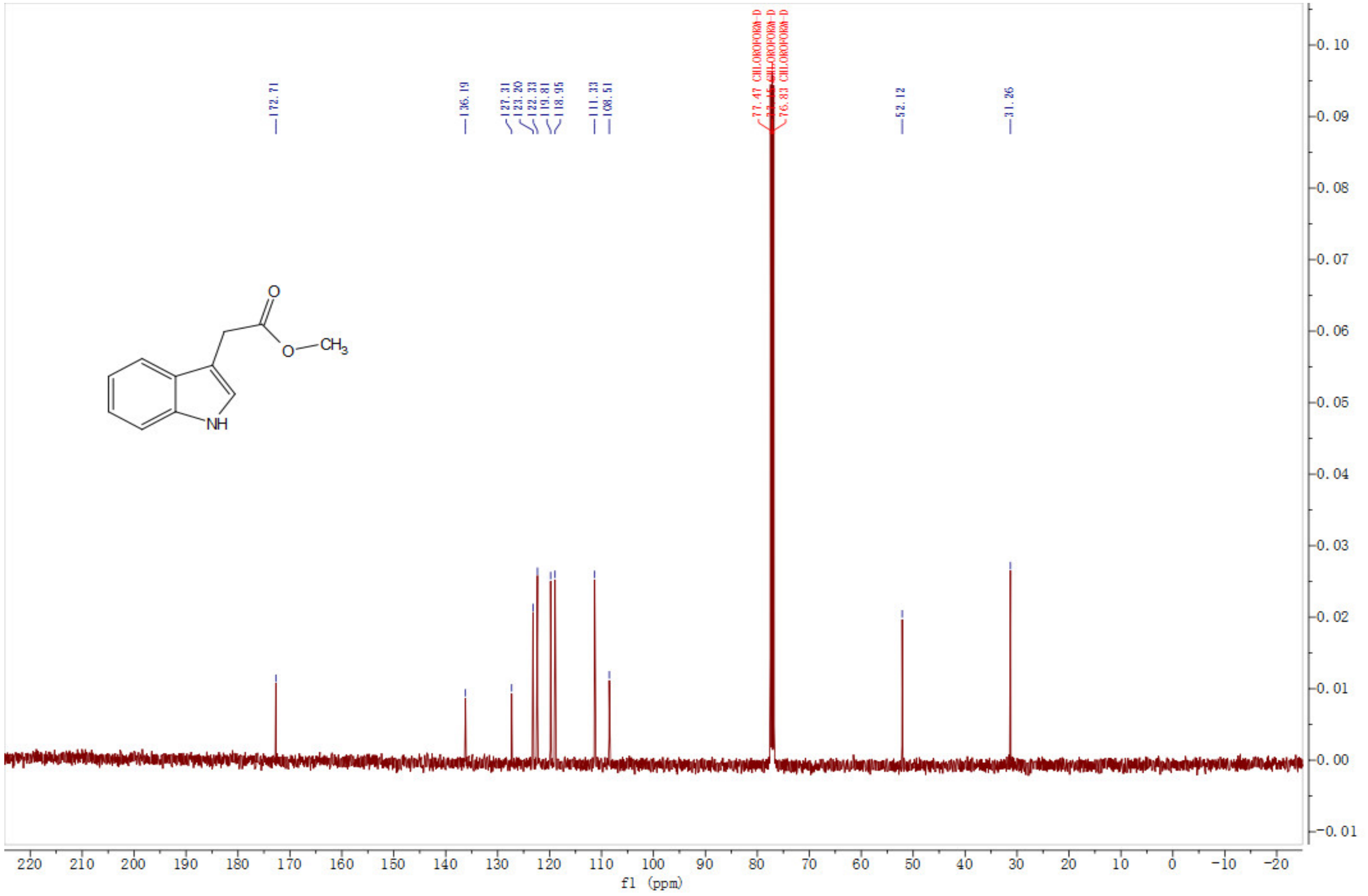

Figure S3. ^{1}H NMR of compound 2

Dibutyl phthalate

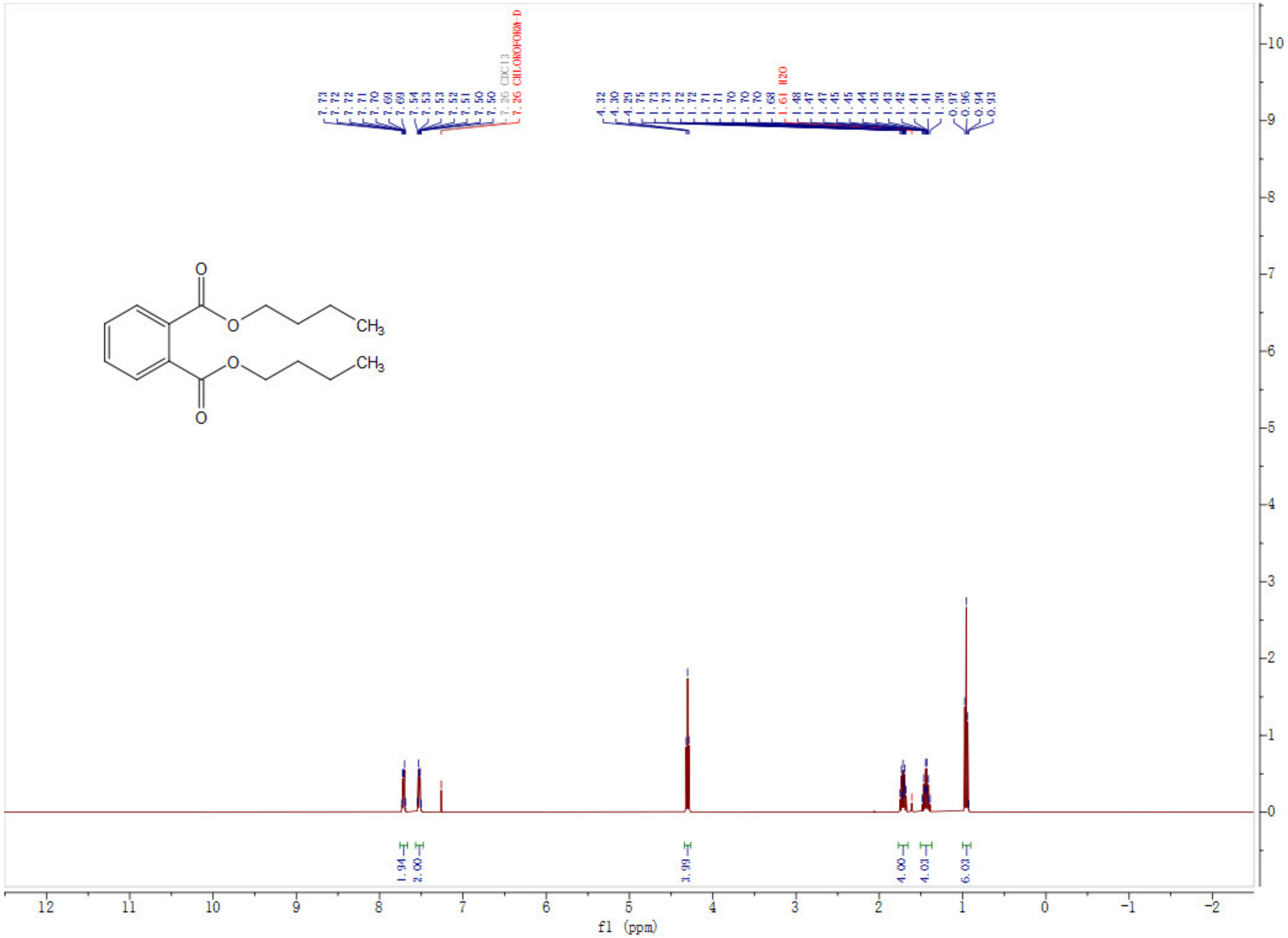

Figure S4. ^{13}C NMR of compound 2

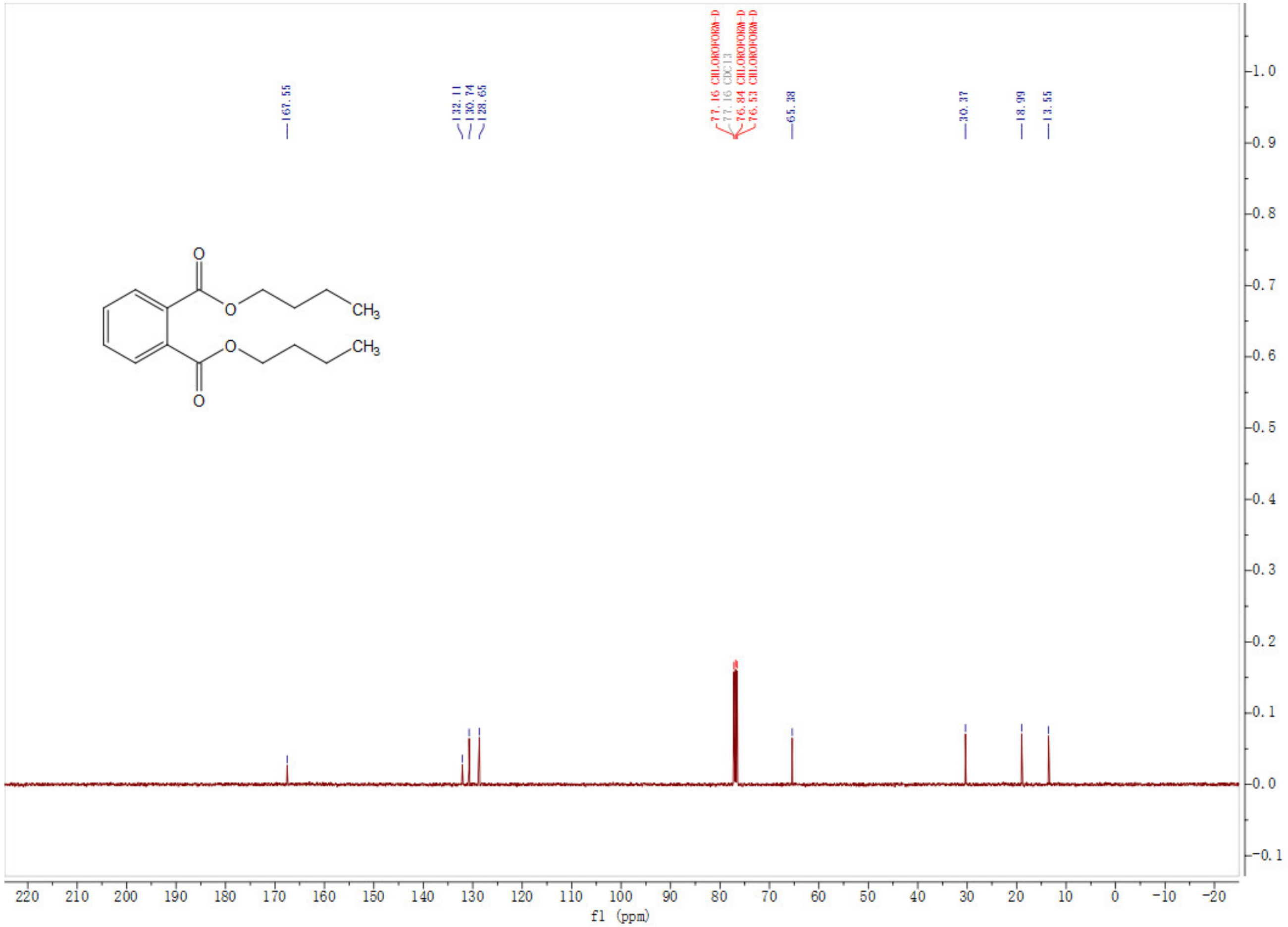

Figure S5. ^{1}H NMR of compound 3

Daidzein

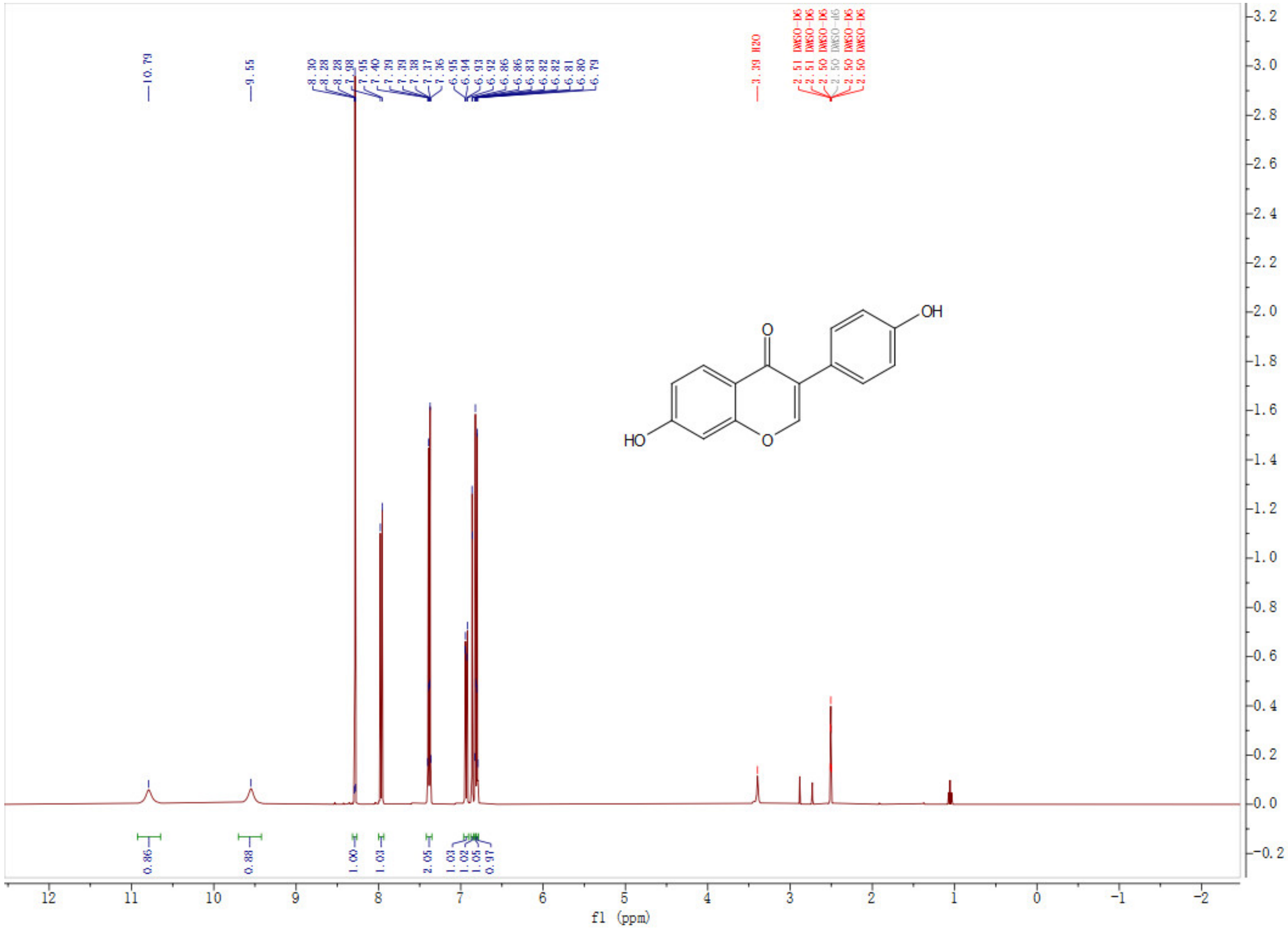

Figure S6. ^{13}C NMR of compound 3

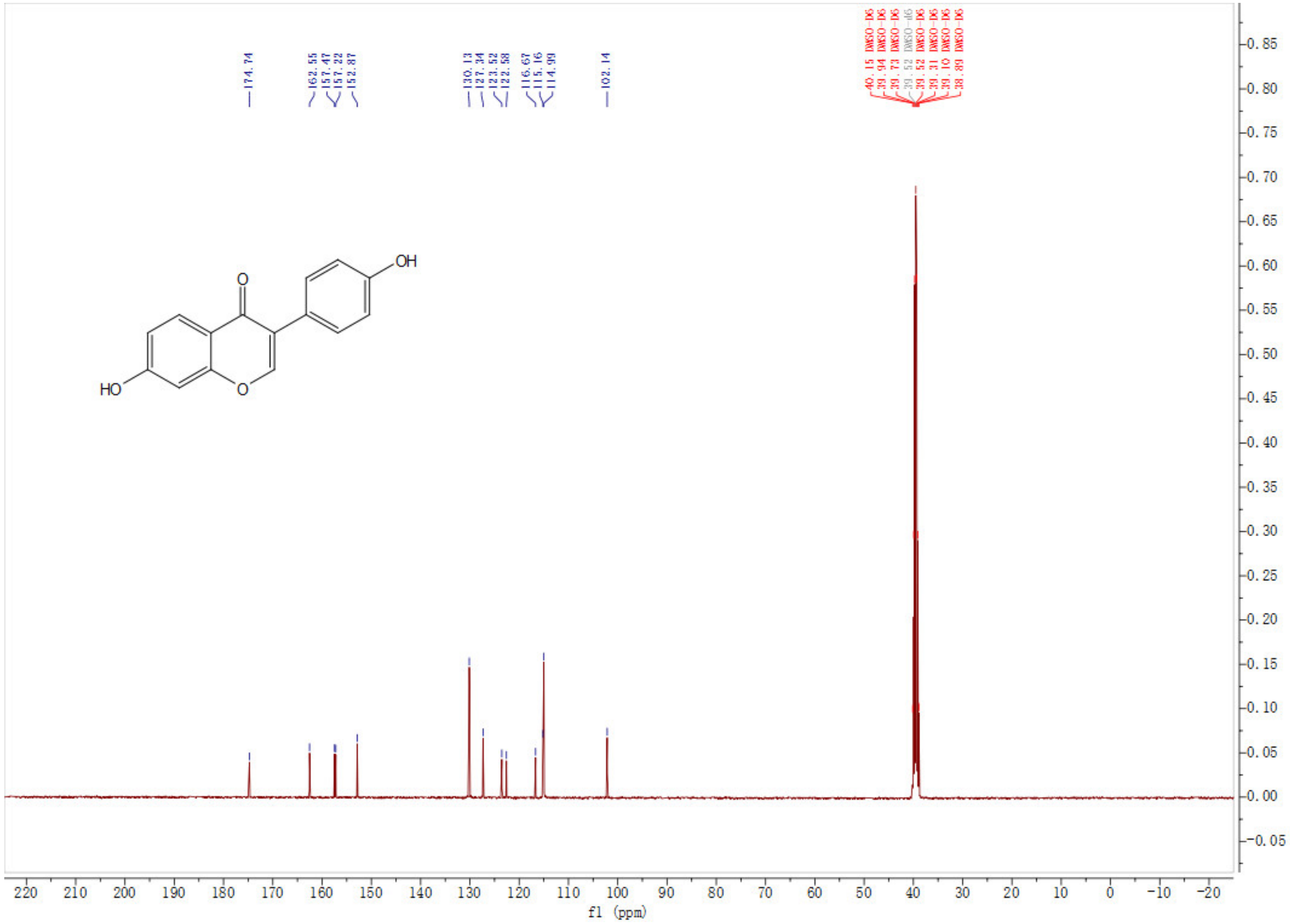

Figure S7. ^{1}H NMR of compound 4

Maculosin

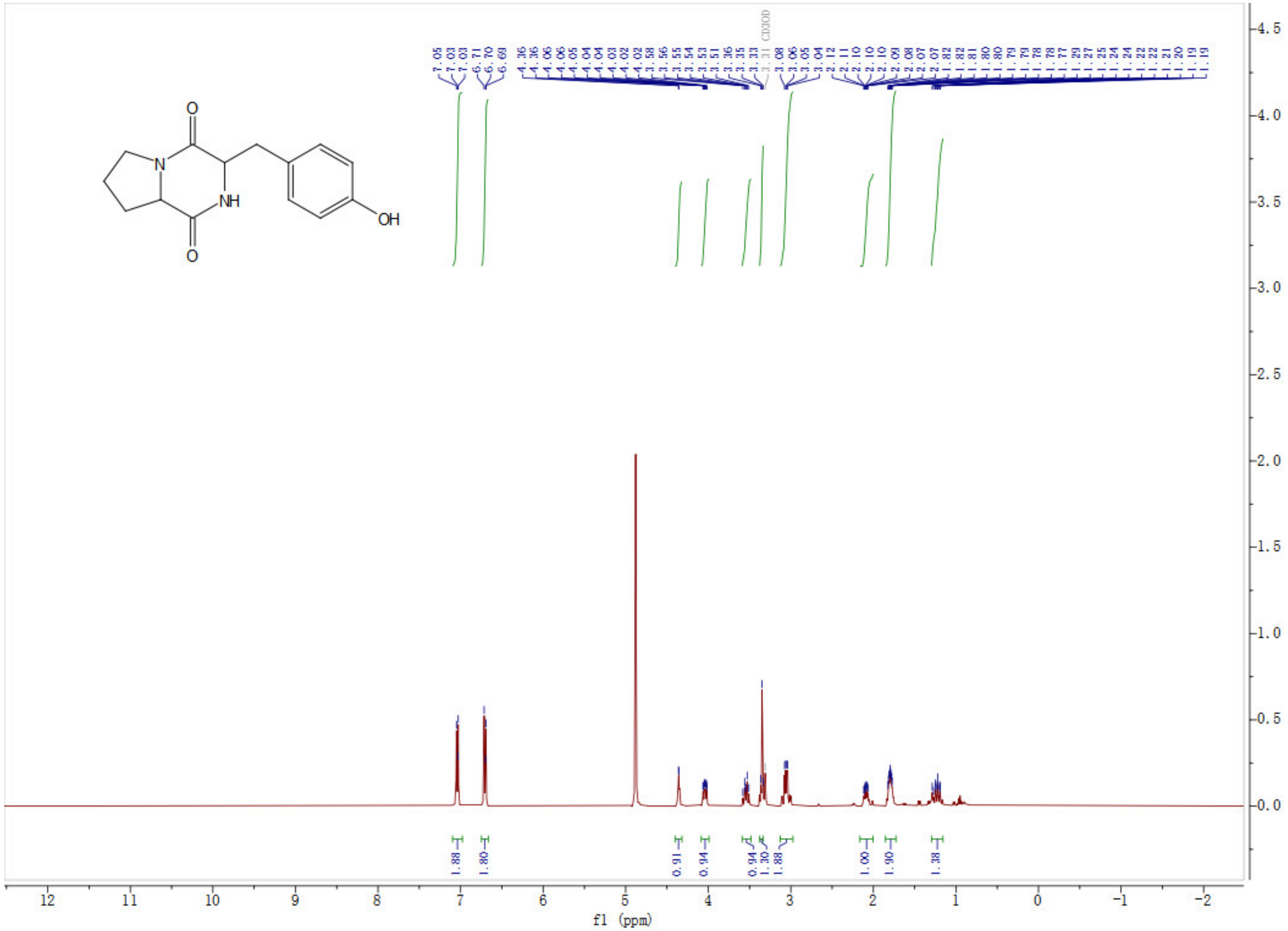

Figure S8. ^{13}C NMR of compound 4

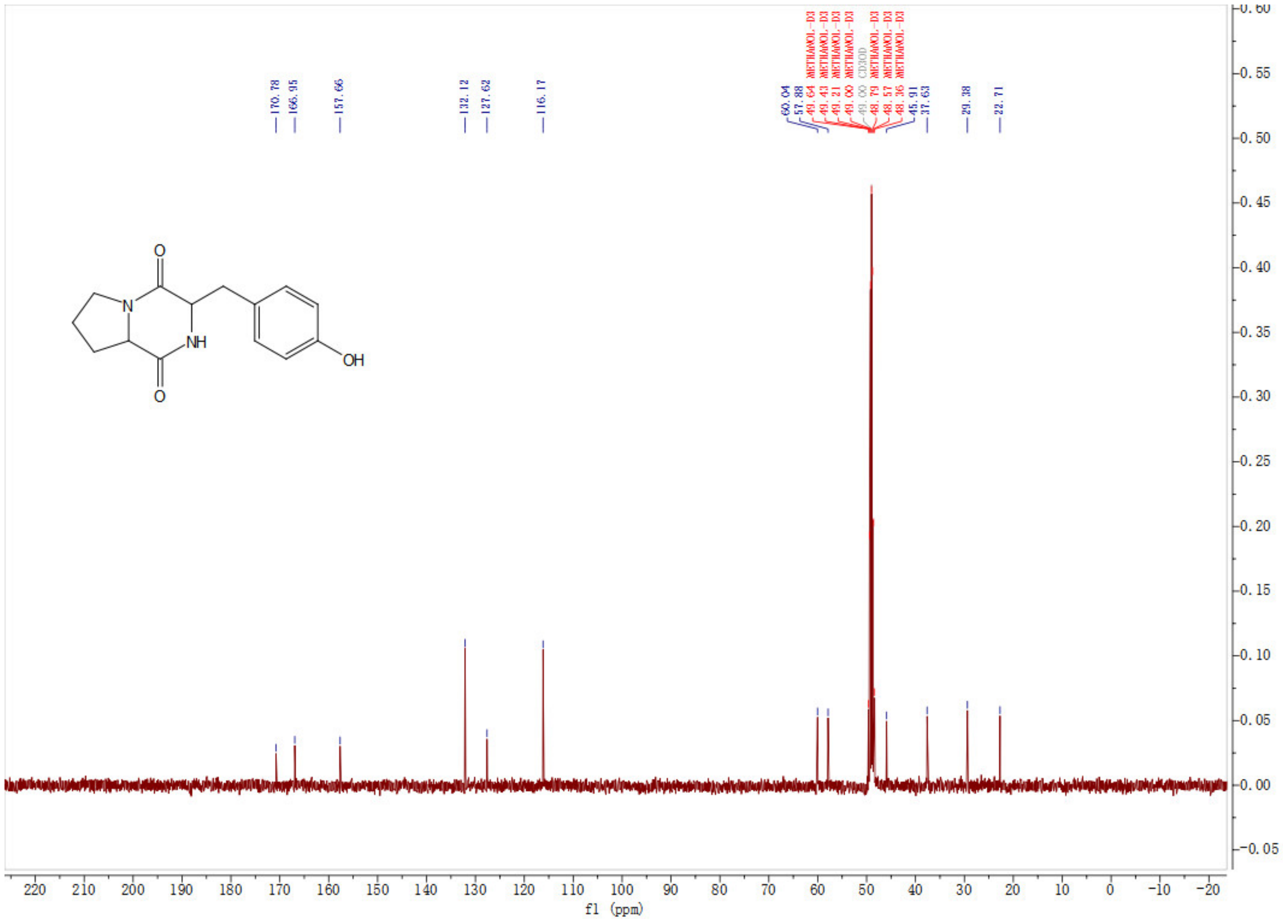

Figure S9. ^{1}H NMR of compound 5

N-Acetyl-L-tryptophan

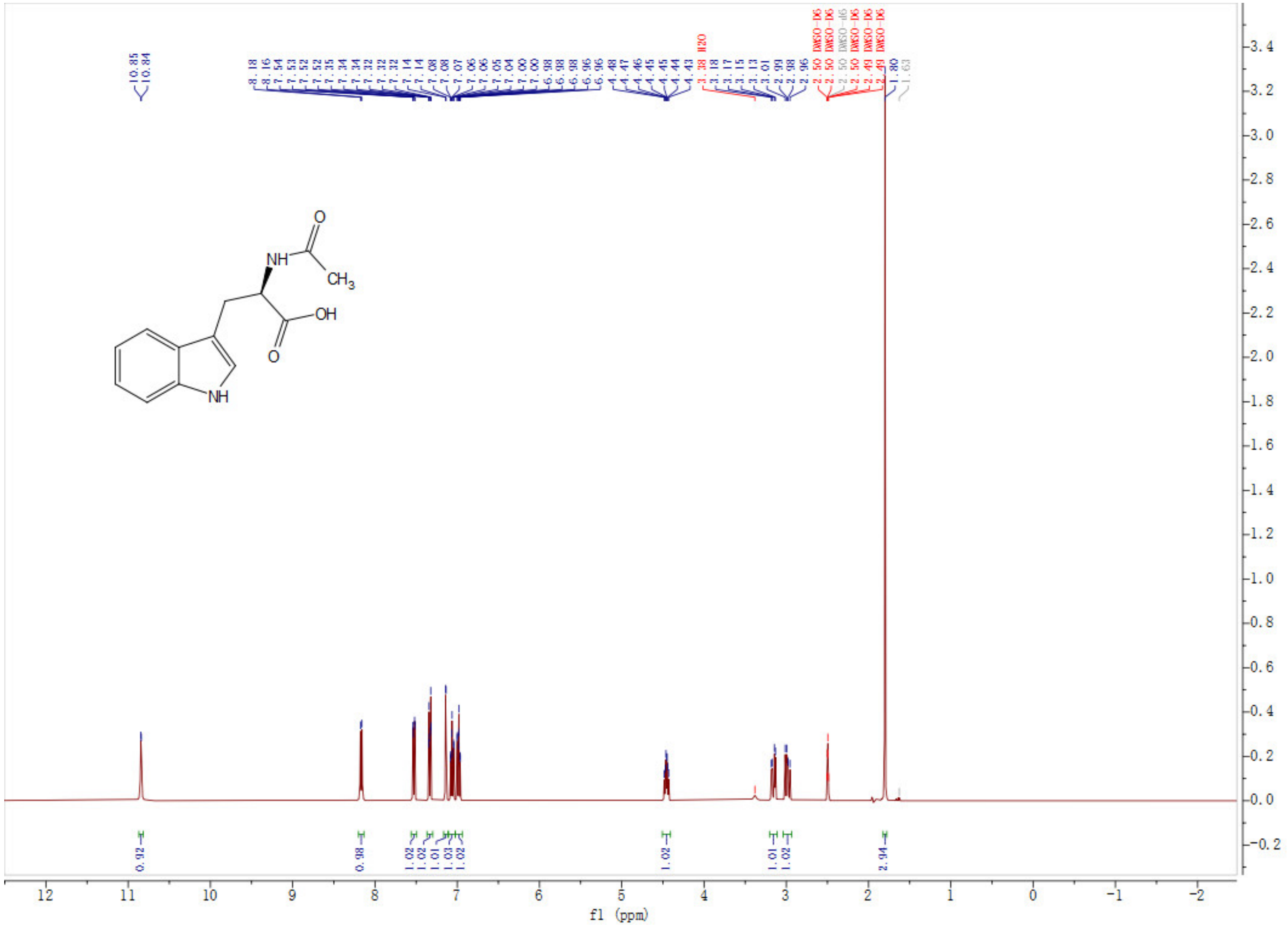

Figure S10. ^{13}C NMR of compound 5

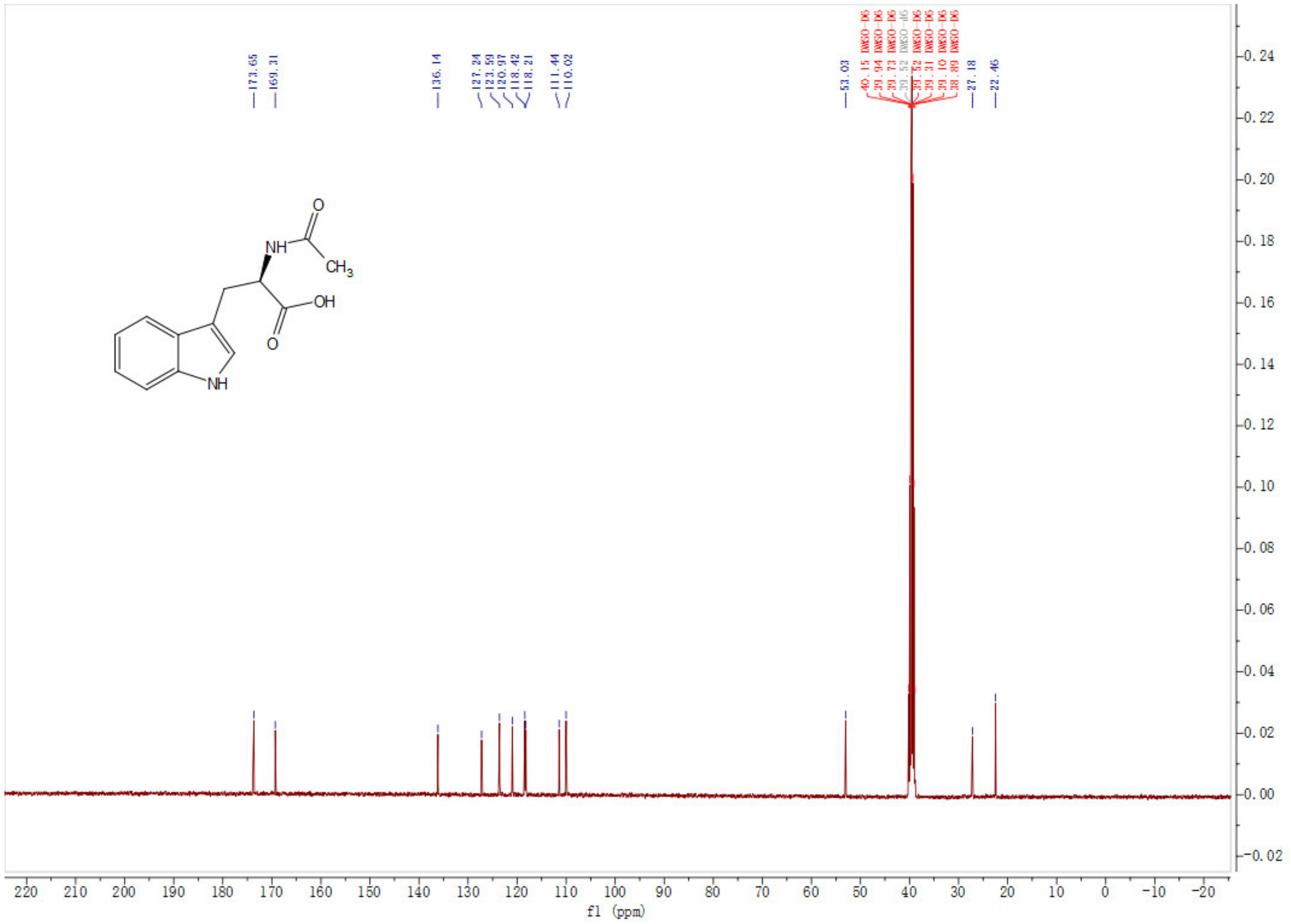

Supplement: Supplementary file 1 [file ijms-25-04611-s001.zip › ijms-2930319-supplementary.pdf]
